# Thermo-Oxidative Decomposition and Ageing of Polymer/POSS Hybrids and Nanocomposites—Failure Predictions and Lifetime Design for Circular End-of-Life Planning

**DOI:** 10.3390/ma19010095

**Published:** 2025-12-26

**Authors:** Tomasz M. Majka, Artur Bukowczan, Radosław Piech, Krzysztof Pielichowski

**Affiliations:** 1 Department of Chemistry and Technology of Polymers, Faculty of Chemical Engineering and Technology, Cracow University of Technology, ul. Warszawska 24, 31-155 Kraków, Poland; 2 Interdisciplinary Center for Circular Economy, Cracow University of Technology, ul. Warszawska 24, 31-155 Kraków, Poland; 3 CUT Doctoral School, Faculty of Chemical Engineering and Technology, Cracow University of Technology, 31-155 Kraków, Poland

**Keywords:** polymer nanocomposites, POSS, hybrid materials, ageing, thermo-oxidative degradation, accelerated weathering

## Abstract

In recent years, hybrid polymer/POSS (Polyhedral Oligomeric Silsesquioxane) systems have attracted particular attention, combining the advantages of organic and inorganic components. This paper reports on the thermal and thermo-oxidative degradation and weathering processes of these materials, as well as their impact on mechanical, chemical, and morphological properties. The paper discusses the physical and chemical changes occurring during degradation, the mechanisms of autoxidation, and the influence of environmental factors such as UV radiation, temperature, and humidity. Particular attention is paid to the role of POSS nanoparticles in polymer stabilization—their barrier function, free radical scavenging, and oxygen diffusion limitation. Methods for analyzing ageing processes are presented, including thermogravimetry coupled with infra-red spectroscopy (TG-FTIR), mechanical property testing, and yellowness index assessment. Material durability prediction models and their importance in designing composite lifespans in the context of the circular economy are also discussed. It is demonstrated that the appropriate type and concentration of POSS (typically 2–6 wt.%) can significantly improve polymer composites’ resistance to heat, radiation, and oxidizing agents, extending their service life and enabling more sustainable lifecycle management of products.

## 1. Introduction

Polymer (nano)composites are an important class of materials that have found various applications, mainly in construction and food packaging [1,2]. They constitute a separate class of filled materials in which at least one dimension of the reinforcing phase is in the nanometer range, usually up to 100 nm [3,4,5,6]. However, polymer-based nanocomposites have been struggling to conquer larger volume market shares ($12–14 billion in 2024 compared to a global composites market estimated at $120–130 billion in the same year [7]), while some weaknesses of nanomaterials, like possible detrimental health/environmental effects and insufficient or not verified long-term stability for out-door applications, are slowing down this development [8]. In general, polymer composites suffer from thermal decomposition through, e.g., random chain scission or chain-end depolymerization leading to reduction in molecular weight and worsening of mechanical properties. Photodegradation and photooxidation under UV radiation can cause crosslinking effects and a surface defect. In polymers containing ester or amide bonds, hydrolysis in the presence of water is also significant, leading to chain breakdown and reduced strength. Moisture and chemicals act as plasticizers (lowering T_g_ and softening the matrix), causing swelling, microcracks, and fiber separation at the interface, which significantly reduces the stiffness, strength, and dimensional stability of the composite materials. Furthermore, differences in the thermal expansion coefficients between the fibers and the matrix, combined with moisture absorption and ageing, lead to thermal stresses, degradation of the fiber-matrix interface, and delamination. The viscoelastic nature of the macromolecular matrix contributes to the long-term instability—creep and stress relaxation cause time-dependent deformations and a gradual reduction in the load-bearing capacity of the composite elements [9]. Some nanoparticles can increase the thermal stability of polymer matrices, enabling their use at higher operating temperatures, or alternatively, they can be used longer at a particular temperature and show a longer lifetime [10,11,12]. Recently, a lot of attention has been paid to hybrid materials, in which there is a covalent bond between the components that are often of different nature, i.e., of organic and inorganic origin. In this way, unique properties of the (nano)hybrids can be achieved that have not been observed in the systems where the components were physically mixed [13,14,15]. In recent years, polyhedral oligomeric silsesquioxanes (POSS) have become one of the most studied nanoparticles in the field of hybrid materials and polymeric nanocomposites [5,16,17,18,19,20,21]. POSS structures with formula RSiO_1.5_, (where R is a hydrogen atom or an organic functional group such as an alkyl, acrylate, hydroxyl, or epoxide), having diameters of the cage ranging from 1 to 3 nm, allow for various chemical modifications. POSS’s unique hybrid structure, containing an inorganic core cage built by Si-O linkages and organic functional groups along its contour, makes it possible to form covalent bonds with functional groups of different macromolecules. POSS nanoparticles were found to enhance various properties of (nano)composites: from mechanical reinforcement, through thermal and UV stability, up to biocompatibility in polymeric biomaterials [22,23,24,25,26,27,28]. In the field of thermal properties, incorporating POSS typically leads to an increase in the glass transition temperature (T_g_), changes in the crystallinity of soft segments, and modification of the order-disorder transition. The effect depends on the type and functionalization of POSS—crosslinking particles restrict chain mobility, while inactive ones can have a plasticizing effect. Importantly, the appropriate selection of POSS type enables precise shaping of the thermal and mechanical properties of polyurethanes [29,30,31,32]. The incorporation of silsesquioxane moieties into polymers in the form of cross-linking agents (as exemplified by polyurethanes) leads to modifications in local molecular interactions, segmental mobility, and molecular topology [12,33,34,35,36] and was found to strongly influence the thermal decomposition routes [37].

This paper discusses the thermal and thermo-oxidative degradation and ageing of polymer nanocomposites and hybrids containing POSS particles. Physical and chemical changes that occur during decomposition, the impact of POSS nanoparticles and the potential applications and research opportunities associated with this promising group of composite hybrid materials are presented.

## 2. Thermo(Oxidative) Degradation of Polymer Nanocomposites

The study of polymer degradation under conditions specifically designed to accelerate the process, along with the extrapolation of the obtained kinetic parameters to milder conditions for the purpose of predicting service life, has attracted considerable interest. The successful development of such empirical and statistical models, as well as theoretical ones, is of significant commercial and practical importance. These models are typically used both for planning the economical replacement of materials before catastrophic failure occurs and for avoiding premature replacement. They can also be employed in developing specifications for quality assurance and control tests of polymer nanocomposites with excellent processing and performance properties [38,39,40,41,42,43].

However, it cannot be expected that simple kinetic models will be able to describe the complex processes occurring in these condensed-phase systems, since predictions derived from such artificial ageing experiments often turn out to be inaccurate [44].

The mechanism and rate of heterogeneous reactions depend on physical factors such as phase transitions, external conditions, and surface properties of the solid phase, which occur within a narrow temperature range. The reaction rate in individual phases may be limited by viscosity and diffusion, and often also by reversibility and cage effects that influence the reaction mechanism. As the reaction temperature changes, the rate-determining step may also change, meaning that not only the values of the kinetic parameters vary, but the form of the kinetic equations themselves may differ across temperature ranges. Extrapolating kinetic parameters over such temperature ranges may therefore lead to wrong predictions [45].

### 2.1. Physical and Chemical Changes During Polymers Degradation

**Figure 1:**
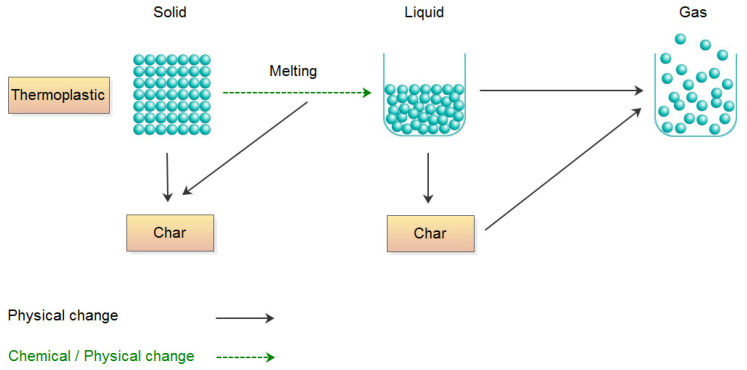
Physical and chemical changes during the thermal degradation process of the polymer matrix.

The lifespan of polymer matrices is limited by their degradation, which may be caused by various environmental factors, including temperature, radiation, mechanical stress, humidity, contaminants, and microorganisms. Thermal degradation is an undesirable process in most applications of polymer composites, as it typically leads to changes in the chemical and physical structure of the matrix–nanofiller system. Progressive degradation of the composite may result in the loss of many valuable properties, such as color [46], impact resistance [47], mechanical strength [48], and molecular weight [46]. Degradation processes are difficult to predict not only because of the aforementioned factors but also due to complex phenomena that are hard to describe, such as diffusion processes, nanofiller interactions, and material morphology. The physical behavior of polymer composites at high temperatures depends on the degree of crystallinity of their macromolecular matrix. In the case of nanocomposites with crystalline matrices, a distinct melting temperature can be observed [46,47,48,49,50,51]. Moreover, many polymer matrices do not reach a viscous state because they undergo thermal degradation before the material has time to melt (Figure 1). Typical polymer materials are only partially crystalline and exhibit a well-defined melting temperature. Due to the structure of thermosetting or cellulosic materials, a change in physical state below their thermal decomposition temperature is not possible. In such nanomaterials, no significant physical transitions occur before decomposition; however, in cellulosic nanomaterials, desorption of adsorbed water always takes place during heating. Depending on whether the water is physically or chemically adsorbed, the temperature and rate of desorption will differ for a given system. Physical desorption of water begins at temperatures slightly below the boiling point of water, and many thermosetting or thermoplastic composites produce charred residues during thermal decomposition. The structure of the char layer has a significant impact on the subsequent course of thermal degradation. Chars (of carbonaceous or mineral–carbonaceous nature) play an important role in stabilizing the degradation process of the polymer matrix. For this reason, properties such as adhesion, cohesion, continuity, density, oxidation resistance, permeability, and thermal insulation capability are crucial factors [52]. Porous char layers may act similarly to nanofillers, as they can effectively limit the heat flux from the gaseous combustion zone to the condensed phase behind it, thereby slowing down the thermal degradation process [53]. Phenomena related to the formation and structure of the char layer are therefore crucial in the design of new polymeric materials with enhanced thermal stability. However, in addition to the processes occurring in the condensed phase, external factors, such as the presence of oxygen or low-molecular-weight compounds, e.g., HCl, may play a significant role in initiation/acceleration of the thermal degradation processes.

The thermal degradation of polymer nanocomposites could begin by oxidative processes or simply by the action of heat or irradiation. In many cases, the thermal degradation processes are accelerated by oxidants. Stuetz found that oxygen can penetrate down to at least 10 nm below the surface of polyolefin [54]. Another study of thermal degradation made by Brauman suggests that the thermal decomposition of poly(methyl methacrylate) is not considerably affected by the presence of oxygen [55,56]. The results of these studies indicate that the effect of oxygen on thermal degradation processes can vary depending on the polymer type and experimental conditions. To better understand the mechanisms underlying these differences, further analysis focused on determining the intrinsic factors affecting the thermal stability of polymeric materials is required.

Kashiwagi, in turn, observed that the thermal and thermo-oxidative degradation of thermoplastic materials is influenced by a number of factors. Among these are molecular weight, weak bonds, prior thermal damage, and the presence of primary radicals. Kashiwagi also suggested that the effect of oxygen on thermal decomposition depends on the polymerization mechanism. His research led to the development of models describing the kinetics of random scission of polymer chains during thermal degradation, decomposition, and destruction of materials [57].

### 2.2. Oxidation Schemes

Oxidation is one of the most important degradation processes. Hoffman demonstrated that the ageing of natural rubber is influenced by its oxygen absorption capacity [58]. Since then, the oxidation of polymeric materials has become the subject of intensive research [59,60]. Oxidation may occur at any stage of the life cycle of polymer nanocomposites—during the storage of the polymer matrix, throughout processing, as well as during the service life of finished composite components. Polymer degradation results in the formation of numerous oxidation and thermo-oxidation products, such as acids, alcohols, aldehydes, esters, ketones, lactones, peracids, peresters, and peroxides [61,62,63]. At room temperature, oxidation of polymer matrices is generally a slow process. However, in the presence of impurities, or when the polymer matrix (or other compounds within the composite system) is highly branched, contains functional groups, or unsaturated bonds, the rate of oxidation and auto-oxidation increases significantly. During processing operations such as extrusion, injection or blow molding, and compression molding, peroxide radicals are formed through reactions with oxygen under conditions of high temperature and/or shear stress. When the polymer matrix is exposed to oxygen, a small concentration of hydrogen peroxides gradually forms over time. However, once a certain concentration threshold is reached, oxidation reactions accelerate rapidly [64]. The mechanism of auto-oxidation, based on the chain reaction theory involving free radicals, is presented in Table 1.

**Figure 2:**
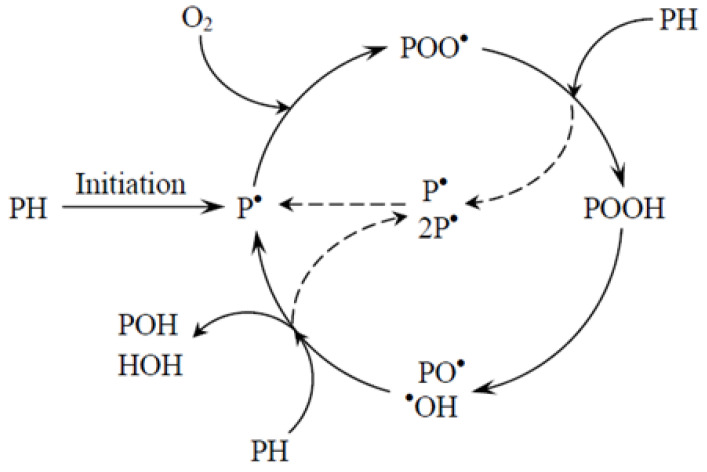
The autooxidation cycle of the polymer matrix.

The fundamental stages of the process include radical initiation, propagation (reaction spreading), chain branching, and termination. At relatively high degrees of oxidation, secondary reactions may become more significant, since applying this model to oxidation in the solid state of polymeric materials assumes that oxidation proceeds uniformly throughout the macromolecular structure [65,66]. The initiation reaction represents the primary stage, during which an alkyl radical (*P^●^*) is formed. It should be noted that during polymerization, catalysts, radical initiators, impurities in the monomers, and trace amounts of oxygen can react to form peroxy radicals (*POO^●^*). These radicals abstract a hydrogen atom from the polymer chain, forming an alkyl radical and hydrogen peroxide (hydroperoxide). The peroxides formed during polymerization decompose under heating or irradiation, producing free radicals that can initiate the auto-oxidation process [67]. Depending on the reaction temperature and the concentration of hydrogen peroxides, their decomposition may proceed via a homolytic or bimolecular pathway [68]. Impurities generated during the production and processing of polymer composites have a significant influence on the initiation rate and the overall rate of oxidation. The course of the reaction also depends on the structure of the radicals formed and the oxygen pressure [69]. The resulting peroxy radicals can abstract a hydrogen atom from the polymer chain, leading to the formation of hydrogen peroxide and a new macroradical, which in turn can initiate another propagation cycle. The hydrogen abstraction by a peroxy radical requires the cleavage of a C–H bond. This reaction is characterized by a high activation energy and constitutes the rate-determining step of the auto-oxidation process. Since the formation of hydrogen peroxides requires high activation energy, an increase in oxidation temperature accelerates the reaction and increases the number of propagation cycles [70]. Chain branching reactions occur when peroxides undergo thermolysis, producing alkoxy and hydroxyl radicals. These radicals can then abstract hydrogen atoms from the polymer chain, leading to further branching of the reaction. The bond dissociation energies of O–H in alcohols and water are approximately 435 kJ/mol and 500 kJ/mol, respectively, while the C–H bond energies in polymer chains are lower, typically below 417 kJ/mol [71]. Therefore, alkoxy and hydroxyl radicals can easily abstract hydrogen atoms from the same or neighboring polymer chains. Termination of the propagation cycle occurs when two radicals combine to form non-radical products. When the oxygen pressure is sufficiently high, termination reactions occur almost exclusively through the recombination of peroxy radicals [72]. If one of the thermolabile peroxy radicals is a primary or secondary radical, the resulting tetraoxide decomposes into an alcohol, a carbonyl compound, and a molecule of oxygen [73], as illustrated in Figure 2.

These reactions influence on the mechanical strength, chemical resistance, and toughness of the polymer composites and could also be the main reason for product discoloration. All of these presented mechanisms could bring about the premature end to the service life of the polymer nanocomposites.

## 3. Ageing and Weathering of Polymeric Materials

With the advancement of science and technology and the growing understanding of nanotechnology, there has been an increasing demand for nanomaterials with higher thermal resistance and favorable thermal properties. The behavior of a material during service under outdoor conditions is a key factor determining the commercial applicability of end products. The service life of a composite at a given operating temperature is often assessed based on the time required for a selected property of the material to degrade to a critical value [10,74]. As ageing progresses, the properties of nanomaterials gradually change until the material loses the characteristics necessary to fulfill its intended functions. The lifespan of a nanocomposite component is of crucial importance in many applications, making the prediction of its durability essential. Comparative forecasting of the full-service life of polymer composites is often based on accelerated ageing tests conducted at regular intervals. This approach enables rapid detection of changes in the bulk properties of the material [75,76,77,78,79,80,81]. The behavior of polymer nanocomposites under natural and artificial conditions is determined by a variety of factors. Complex polymer products are typically exposed to different, often extreme, atmospheric conditions, including solar radiation, temperature fluctuations, and precipitation such as rain or snow. Experiments are frequently carried out under artificially simulated conditions to shorten testing time; however, it should be noted that the results may differ from those obtained under natural conditions due to interactions between atmospheric factors and stabilizing additives present in polymer nanomaterials [82]. Chemical and physical ageing mechanisms of polymers may occur simultaneously. The former primarily affects the molecular structure of the polymer, while physical processes are associated with the diffusion of additives toward the surface [83]. Atmospheric ageing of polymer composites leads to photo-oxidation and crosslinking reactions, which cause significant changes in the molecular structure of the polymer matrix. Under environmental conditions, degradation involves the gradual leaching of inorganic components and plasticizers, whereas under thermal ageing conditions, the main degradation mechanism is the reorganization and aggregation of molecular chains as a result of annealing [84,85,86,87]. Thermal and accelerated atmospheric ageing processes of polymer nanocomposites, aimed at predicting their durability, usually involve practical tests such as: assessment of yellowing or discoloration, analysis of thermal decomposition kinetics, evaluation of mechanical and morphological changes after heat treatment, determination of volatile products using TG-FTIR or TG-MS, and measurement of the heat deflection temperature along with other thermal resistance parameters.

Outdoor-service polymer nanocomposites experience simultaneous chemical and physical ageing (weathering), so their durability is commonly evaluated via accelerated ageing tests that monitor how key properties degrade over time. This leads directly into the next subsection on Color Characterization and Yellowness Index Determination, which treats yellowing/discoloration as a practical, sensitive indicator of progressing (thermo-)photo-oxidative ageing.

### 3.1. Color Characterization and Yellowness Index Determination

Photo-oxidation of organic materials is the main cause of their irreversible degradation. It is responsible for the loss of physical properties in polymer composites and rubber, as well as for the deterioration of food quality [88]. In most polymeric materials, photo-oxidative degradation can be induced by UV radiation or catalytic processes, and its rate increases at elevated temperatures [89]. The harmful effects of atmospheric ageing of polymer matrices are attributed to a complex set of processes, in which the combined influence of UV radiation, light, heat, and oxygen plays a dominant role. Due to the presence of oxygen in the atmosphere, a purely thermal effect is practically impossible, meaning that the process is thermo-oxidative in nature [90]. There are many different mechanisms of polymer degradation, which are similar in that they all involve chemical reactions leading to bond cleavage. These mechanisms may resemble those presented in Table 1. Photo-oxidative degradation of polymer hybrids, involving processes such as chain scission, crosslinking, and secondary oxidation reactions, occurs through radical pathways similar to those of thermal oxidation [91]. Among the many mechanisms proposed to explain the photo-oxidation of polymer hybrids—consistent with observations made for low-molecular-weight compounds—one deserves particular attention. It involves the formation of radicals and their subsequent reactions with oxygen [92]. Internal and external chromophoric groups absorb light and generate low-molecular-weight radicals (*R^●^*) or polymer macroradicals (*P^●^*). This reaction can be initiated by physical factors (such as heat or mechanical stress) or by chemical factors. The most important initiator in the photo-oxidation process is hydrogen peroxide, similar to the mechanism of thermo-oxidative degradation. Regardless of the primary mechanism of radical formation, reactions with oxygen lead to the formation of peroxides, which serve as key intermediates in the polymer oxidation process. The propagation stage can be divided into several steps, including:

- Abstraction of a hydrogen atom from the same or another molecule by an alkoxy radical, leading to the formation of hydroxyl groups.
- Abstraction of a hydrogen atom from the same or another molecule by an alkylperoxy radical, resulting in the formation of a hydroperoxide group.
- Further reactions of low-molecular-weight radicals and alkyl radicals in a chain process similar to hydrogen abstraction.
- Reactions of macroradicals with oxygen, leading to the formation of peroxy radicals.
- Photodecomposition of hydroperoxide groups, producing alkoxy, peroxy, and hydroxyl radicals within the polymer structure.
- Cleavage of alkoxy radicals resulting in terminal aldehyde groups and alkyl radicals.

Radicals formed during the degradation of polymer matrices may undergo termination through numerous recombination reactions between two polymer radicals or with other radicals, resulting in the formation of non-reactive products. Understanding how radical reactions proceed during thermal and oxidative degradation is crucial for developing polymeric materials with improved aging resistance. In this context, special attention shall be paid to structural modifications of polymers and additives that can limit the formation or activity of radicals, thereby slowing down the degradation processes.

The yellowing effect caused by thermo-oxidation of PU/POSS composites was studied by Zhao et al. [93]. It was found that the incorporation of moisture-cured polyurethanes with silsesquioxanes containing epoxy substituents delayed the onset of ageing effects from 8.32 h (for the pure material) to 20.78 h (for the hybrids), significantly improving resistance to yellowing.

In another study on the influence of POSS on the behavior of ABS subjected to thermo-oxidative ageing, three types of POSS, aminopropyl isobutyl (APOSS), glycidyl (GPOSS), and trisilanol (TPOSS)—each at 5 wt.%, were used. Colorimetric analysis (CIE LAB) demonstrated that all samples—both pure ABS-g-Ma and the POSS-based nanocomposites—yellowed and darkened at similar rates during thermal ageing. Differences in color change (ΔE) were negligible: TPOSS showed a marginally better result, while APOSS performed the worst. Authors concluded that despite good dispersion and phase compatibility, the incorporation of POSS nanoparticles did not significantly enhance the thermal resistance or color stability of ABS-g-Ma, and the observed effects were minimal and mutually compensating. This is an example of the fact that the addition of POSS does not always improve the properties of all kinds of polymers [94].

Photo-oxidative stability has also been investigated for POSS/polymer composites. Musto et al. [95] used octaglycidyldimethylsiloxy-POSS (OG-POSS) in an epoxy matrix to reduce its sensitivity to UV radiation. It was found that the addition of 20 wt.% POSS nanoparticles prevented microcracking on the surface of the composites, which was explained by the barrier effect of the inorganic layer. However, it should be noted that such a large addition of POSS may be difficult to reproduce on a larger scale due to its high cost. Furthermore, such a large addition of POSS may cause agglomeration in the material, which will negatively affect its mechanical properties.

In another study, the authors used different types of POSS nanoparticles to examine their influence on the oxidative stability of polystyrene [47]. It was discovered that the addition of silsesquioxane units can effectively protect the PS surface from UV radiation due to the presence of the inorganic silica core. Moreover, open-cage POSS with hydroxyl groups exhibited the most effective barrier properties.

It naturally precedes the next subsection on structural changes during thermal and accelerated ageing by shifting from optical, colorimetric indicators of degradation to the underlying chemical/structural and morphological transformations that drive these observable color changes (Table 2).

### 3.2. Structural Changes During Thermal Ageing and Accelerated Weathering

One of the methods for predicting the lifetime of polymeric materials based on thermal ageing is thermal analysis. Thermogravimetry (TGA) is an effective technique for calculating the kinetic parameters of polymer degradation, which can subsequently help in calculating their life cycle. Frone et al. found that branched substituents in the POSS structure enable the formation of a stable ceramic phase during thermal degradation of POSS-modified polyethylene, resulting in improved mechanical properties and increased degradation resistance [102]. In their work, they conducted TGA studies, which showed that POSS completely evaporates without decomposition in an inert atmosphere, while in air it leaves a residue of 20–46%, depending on the heating rate. This behavior indicates a strong cohesive interaction between the organic and inorganic phases in nanocomposites, e.g., polystyrene modified with POSS containing alkyl groups. TGA data, supplemented by kinetic calculations, allowed the determination of the apparent activation energy of degradation. Bianchi et al. showed that in the case of polystyrene modified with methacrylphenyl-POSS (PS/methacrylphenyl-POSS) with a 5% inorganic phase, the activation energy values remained higher than those of the pure polymer over a wide range of conversion degrees, indicating increased resistance to oxidation and thermal bond cracking. A similar evolution of the apparent activation energy for aerobic and anaerobic conditions was also observed by other authors [103], confirming that the addition of POSS limits the propagation of oxidative reactions in the polymer matrix. The increased activation energy values are interpreted as evidence of the stabilizing effect of POSS, resulting from the presence of strong interfacial bonds and the effect of blocking oxygen migration in the composite structure [104]. However, other thermo-analytical techniques enable more detailed tracking of the thermal decomposition stages and provide characteristics of the (volatile) degradation products.

Hence, Lewicki and co-workers conducted TVA degradation analysis to compare the thermal resistance of polyurethanes (PUs) and their hybrids containing POSSePU. Four degradation stages were observed under high-vacuum conditions: loss of water and low-molecular-weight residues, release of entrapped air, primary depolymerization of urethane bonds, and high-temperature rupture of polyol chains accompanied by carbon monoxide emission. The POSSePU hybrids demonstrated significantly higher thermal stability—the onset of primary degradation shifted from ~260 °C (pure PU) to approximately 285–295 °C (hybrid systems), with the best result achieved for a 2% additive. Furthermore, the hybrids released significantly fewer volatile products (~50% decrease), suggesting that degradation is more gentle and leads to the formation of heavier, less volatile chain fragments and limited diffusion of light products through the material [105].

For determining structural changes in composites during thermal or accelerated atmospheric ageing FTIR spectra are the simplest and most effective analytical methods. An example of work using infrared spectra as one of the analytical methods is the study by Dintcheva et al., who analyzed photo-ageing of polystyrene (PS) composites containing 5% of various POSS. The samples were subjected to accelerated UV ageing (UVB lamps, 55 °C, for up to 240 h), and degradation was assessed using FT-IR, UV–Vis, SEM, EDX, and contact angle measurements. Compared with pure PS, composites with POSS exhibited slower growth of carbonyl and hydroxyl bands, a longer oxidation induction period (up to 48 h for open POSS triols), and a lower degree of surface oxidation. POSS did not alter the degradation mechanism but significantly slowed it down. Surface analyses showed that the composites retained increased hydrophobicity after UV irradiation, and EDX analysis revealed an increase in silicon content after ageing, indicating the migration of POSS to the surface and their protective function. UV–Vis spectra showed that POSS absorb radiation at wavelengths of 290–350 nm, acting as effective UV filters, particularly for TSIO- and TSPH-POSS. It was found that all POSS acted as UV filters (in the range of ~290–350 nm), thereby limiting the photodegradation of PS, with the open POSS triplets being the most effective [96].

In another work, the effect of ageing on polypropylene (PP) composites containing various synthetic organosilicon compounds was investigated. Of particular interest were PP/POSS composites. They were subjected to accelerated laboratory ageing in an Atlas Weather-Ometer Ci4000 (Atlas Material Testing Technology, Mount Prospect, IL, USA) apparatus, in accordance with ISO 4892-2 A1. After each exposure stage, the samples were analyzed using FTIR, SEM, and DSC methods. FTIR analysis showed an increase in the intensity of carbonyl, vinyl, and hydroxyl bands compared to pure PP. Additionally, POSS8 and POSS18 slowed the formation of ketones and carboxylic acids—the main oxidation products. This indicates that the addition of POSS to PP resulted in a lower degree of oxidative degradation and a longer induction period of ageing. DSC analysis, on the other hand, revealed a decrease in melting and crystallization temperatures compared to pure PP, which suggests slower destruction of the crystalline structure [97].

Structural changes occurring in composites containing POSS are of crucial importance in space technologies. Bram et al. conducted studies assessing the effect of the type and amount of POSS nanoparticles on the resistance of epoxy resins under simulated space conditions. Outgassing tests showed that the addition of POSS improved material stability, especially in the case of AM-EPOSS (POSS with amino groups), which met all space standards. The EP-EPOSS versions (with epoxy groups) performed worse due to higher polymer chain mobility and greater evaporation of monomer residues. During exposure to atomic oxygen (AO)—the main ageing factor in low Earth orbit—all POSS-containing composites exhibited significantly lower erosion than pure epoxy. The best performance was observed for AM-EPOSS samples, which showed up to 20 times less erosion. This was due to the formation of a self-limiting silicon oxide (SiO_2_) layer that protected the surface from further oxidation [98].

The protective SiO_2_ layer was also the subject of other studies, in which the effect of POSS addition on the thermal ageing of rigid polyurethane foams was investigated. It was found that the presence of POSS did not significantly increase the material’s resistance to high temperatures, as POSS itself begins to decompose within a similar temperature range as polyurethane. Nevertheless, its introduction changed the degradation mechanism—leading to the formation of a greater amount of solid residue containing silicon compounds, which acted as a protective layer in the subsequent stages of combustion [106].

In turn, Dharmaraj et al. performed ageing studies of Ph-POSS@ZIF-8 and Ph-POSS@ZIF-8@PDA@Sponge composites, which demonstrated their high chemical and mechanical durability under harsh environmental conditions. The use of an octaphenyl-POSS nanocage significantly improved the resistance of ZIF-8 to degradation and loss of hydrophobic properties. In chemical tests (acidic, basic, and saline solutions) [100], pure ZIF-8 dissolved rapidly, while Ph-POSS@ZIF-8 maintained structural integrity and hydrophobicity. AAS analysis showed much lower zinc leaching—7.2 ppm (acid), 5.1 ppm (base), compared to 51.8 and 49.2 ppm for pure ZIF-8. In long-term operation tests (25 separation cycles), the composites maintained >90% separation efficiency under neutral conditions and ~85% in acidic and basic environments. SEM observations confirmed the absence of morphological changes [101].

**Figure 3:**
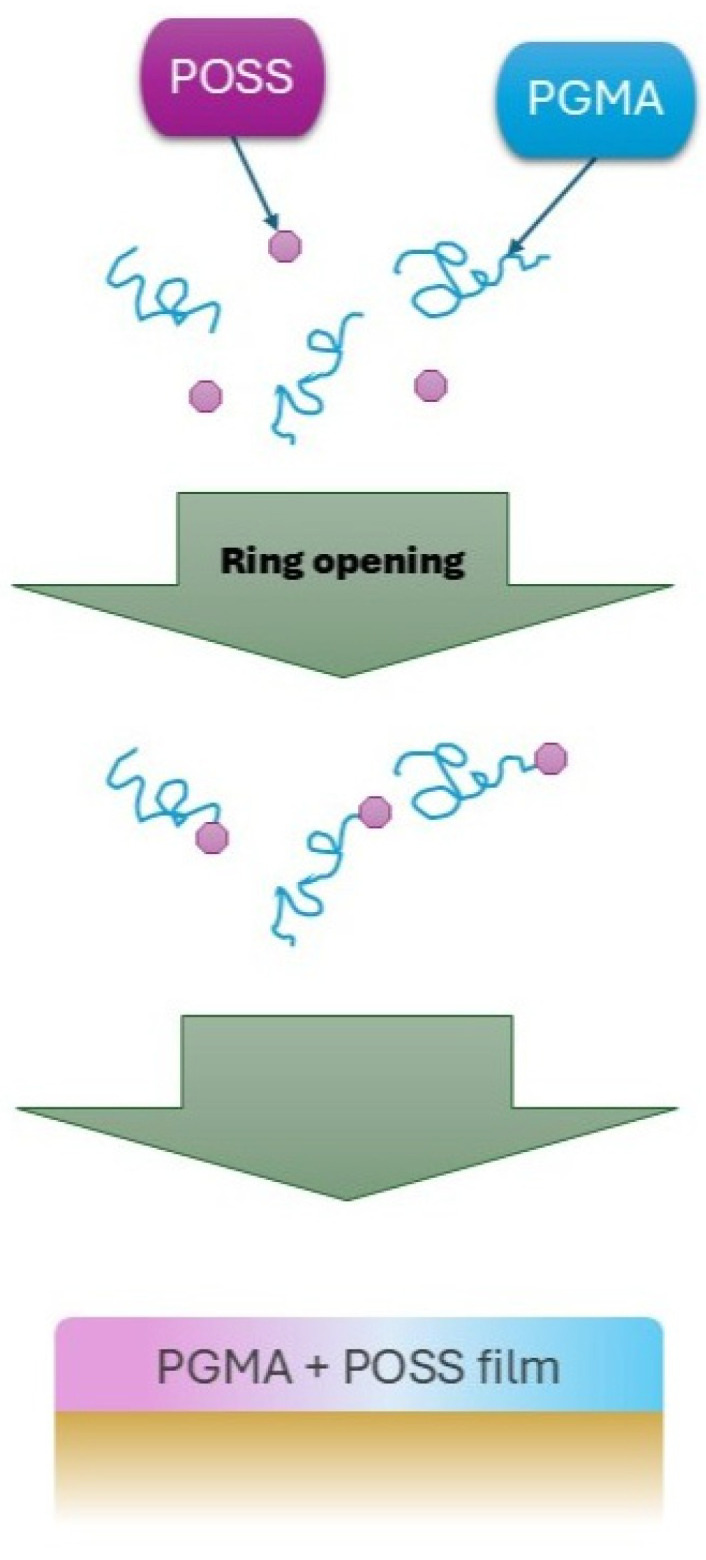
Flow diagram about coating construction at the surfaces of decorative paintings. Figure based on [107].

Noteworthy, polymer/POSS composites have been applied in the protection of artworks. An example is the study by Xiong and Li, in which transparent, hydrophobic protective coatings POSS@PGMA were developed to safeguard decorative paintings against water and light. The coatings were produced via an in situ reaction between poly(glycidyl methacrylate) (PGMA) and POSS, forming a three-dimensional, crosslinked structure strongly bonded to the substrate. Due to the migration of POSS nanocages toward the surface (this was shown in Figure 3), high roughness, low surface energy, and high transparency (>80%) were achieved. The durability of the coatings was attributed to the strong Si–O bonds in the POSS structure, dense PGMA crosslinking, and preserved surface roughness.

Ultimately, the POSS@PGMA composites demonstrated excellent ageing resistance while maintaining high hydrophobicity, transparency, and thermal stability (Tg ~ 113 °C), making them an effective protective material for decorative paintings [107].

These presented structural signatures provide the basis for the next subsection, where the same ageing-induced transformations are translated into changes in tensile/compressive strength and modulus retention.

### 3.3. Mechanical Properties Changes During Ageing

Impact of UV radiation on the mechanical properties of liquid crystalline/POSS composites was described in work by Jang et al. [99]. In this research poly(p-phenylene ben-zobisoxazole) (PBO) was modified with trisilanolisobutyl-POSS and then subjected to tensile and compressive strength tests before and after UV radiation. The protocol of the ageing test consisted of UV radiation under the 400 W lamp with a wavelength range of 300–500 nm for 8 and 16 h. Noteworthy, it was found that already 2 wt.% POSS loads can significantly delay loss in mechanical properties during the ageing process. The obtained results clearly demonstrate that an appropriate type and amount of POSS nanofiller can effectively mitigate the adverse effects of photochemical ageing, thereby improving the durability of polymer materials, especially maintaining their mechanical properties. This phenomenon has also attracted the attention of other researchers, who investigated the influence of POSS fillers on the behavior of various polymer systems subjected to ageing under different environmental conditions.

**Figure 4:**
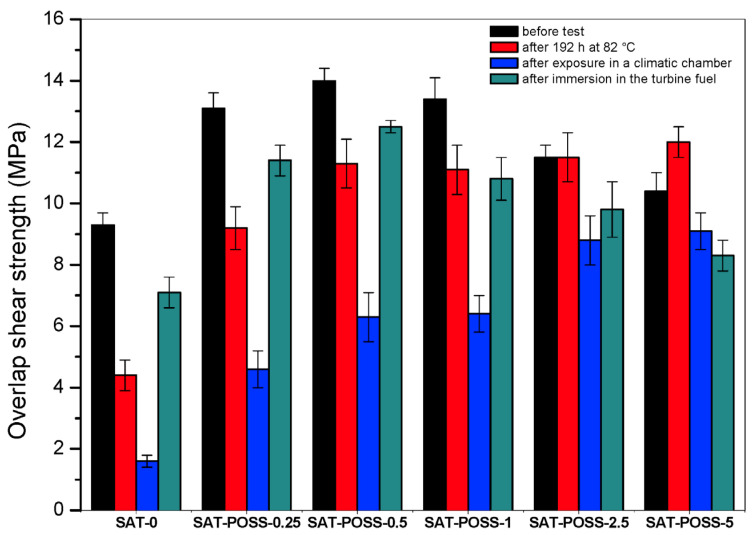
Shear strength test before and after ageing. Reprinted with permission from [108].

Kowalczyk et al. [108] studied the influence of hybrid epoxy acrylate copolymers (EA) on the properties of structural self-adhesive tapes (SATs) after different ageing tests. Significant changes in overlap shear strength after POSS addition (0.25–5 wt.%) were observed (Figure 4).

The effect of POSS nanoparticles on the mechanical properties of polymers during ageing was also described by Zaharescu and co-workers. POSS nanoparticles act as diffusion barriers and free radical traps, thus limiting the worsening of mechanical properties. Composites containing POSS exhibit a slower decline in tensile and compressive strength and a smaller reduction in elastic modulus compared to unmodified materials. This phenomenon results from improved microstructural stability and interfacial reinforcement, which inhibits crack propagation during thermal and radiation ageing. An optimal POSS content (approximately 6 wt.%) promotes the integrity of the polymer network, while an excess leads to agglomeration and local stiffness loss. Overall, POSS extends the service life of polymers by maintaining their mechanical resistance at a higher level for a longer period [104].

This subsection shows that low, optimized POSS loadings can slow the ageing-driven decline of tensile/compressive and shear strength (and modulus) by reinforcing interfaces and hindering oxygen/radical-driven damage, whereas excessive POSS may agglomerate and reduce the beneficial properties enhancements. This sets up the next subsection by linking these macroscopic property-retention trends to the specific ageing mechanisms and formulation factors that control degradation rates.

### 3.4. Lifetime Predictions of Polymeric Materials

One of the drawbacks of composites is their inherent dependence of mechanical properties on time. In applications where a design lifespan of up to 50 years is required—such as in the aerospace industry, bridge structures, or water and sewage systems—it is not feasible to test these materials or structures over such long periods to fully encompass their expected service life. Therefore, there is a strong need to develop accelerated durability assessment methods that allow the prediction of strength and thermal stability changes in polymer composites, ensuring the integrity and safety of structural components. One of the major challenges is determining how a material will behave under real service conditions, which may involve multiple factors such as light, temperature, humidity, atmospheric pollutants, or radiation—some of which depend on geographic location. Ageing tests are often conducted to address three interrelated objectives:

- To conduct scientific research aimed at understanding the mechanisms of polymer degradation.
- To perform comparative tests for selecting materials with the best stabilization effects.
- To predict service life, i.e., to determine how long a given material will retain its properties.

Understanding degradation factors and their mechanisms of action is essential for developing appropriate procedures to evaluate material properties [109,110,111]. One of the key challenges arises from the complexity of polymer nanocomposite composition. The components—which may include more than one type of polymer or other elements such as pigments or nanofillers—are randomly distributed during manufacturing and typically retain a similarly nonuniform distribution after drying or curing, although some systems show a certain degree of dispersion. From an industrial perspective, it is crucial to obtain reliable and practical information in the shortest possible time to enable rapid selection between materials and product prototypes, benefiting both manufacturers and end users. In this context, reliability engineering plays an important role—laboratory tests under high stress can provide failure probability distributions in a short time, unlike tests conducted under natural conditions. However, correlating accelerated ageing with natural exposure is very difficult, as different damage mechanisms may occur in both the composite and the substrate. This limits the usefulness of failure probability distributions for polymer composites. Nevertheless, accelerated tests are widely used to eliminate materials exhibiting weaknesses. Unfortunately, such tests often hinder the introduction of new materials, as they do not allow for the reliable prediction of real-life performance [109,112,113,114,115,116,117,118,119,120,121,122,123,124]. Developing an effective model to describe the degradation of heterogeneous and complex materials requires the application of universal concepts with broad applicability. The proposed approach focuses on the wear-out regime—the phase of material use where damage mechanisms result from long-term, continuous exposure to environmental factors, rather than from design or manufacturing errors [125].

### 3.5. Reliability Engineering

**Figure 5:**
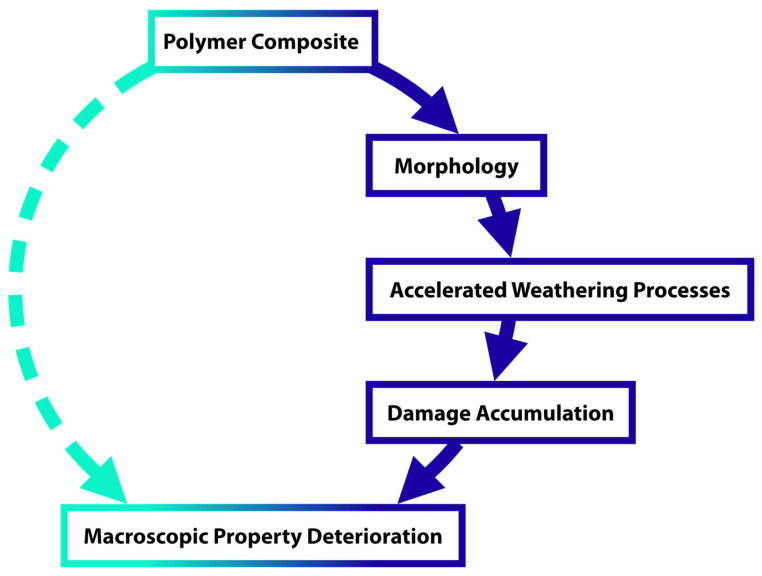
A scheme for overall task of predicting service lifetime of hybrid materials.

The relationship between material composition and operational durability is highly complex, but it can be simplified by breaking it down into component processes. For each key functional property, several stages can be identified that link composition to material performance (Figure 5). Above all, degradation mechanisms should be identified as precisely as possible. For instance, some composites exhibit an initial increase in gloss during early exposure, caused by surface smoothing of the polymer softened by the elevated temperature of the ageing cycle, before the gloss begins to decline due to surface roughness induced by degradation. Isolating such individual processes also allows differentiation between degradation of the composite and possible degradation of the substrate, which may also contribute to the overall material deterioration process. There are other ways to represent this relationship, but any such approach is useful because it allows the problem to be divided into specific research questions, enabling more focused investigation and progress in analysis [126,127,128,129,130,131].

**Figure 6:**
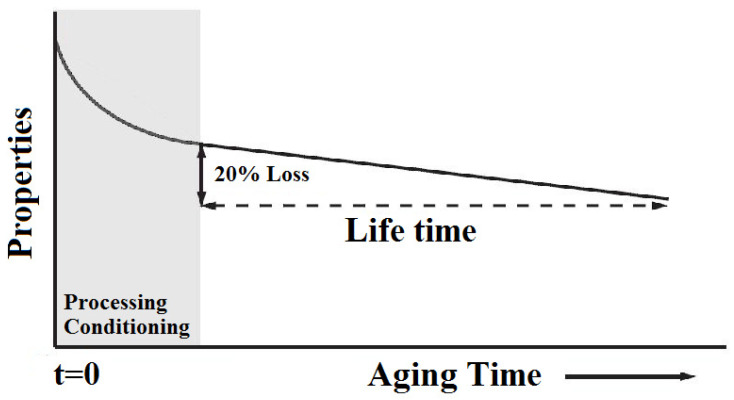
General linear decay of polymer composites’ properties through lifetime.

As already mentioned, this is a conventional approach used in reliability engineering within the composites industry. This method allows for the elimination of materials with inherent weaknesses but has proven insufficient for predicting actual service performance. Extrapolation of results to estimate service life is much more reliable and safer when it is based on fundamental knowledge of degradation processes (Figure 6). These steps can be referred to as a bottom-up approach. If the chemical and physical structure of a material is understood, analytical methods can be used to determine its degradation mechanisms. However, this does not always make it possible to clearly identify which factors are most significant or how they affect material durability. Quantitative estimation of service life requires understanding how damage accumulates and how this knowledge can be applied to calculate the rate of deterioration of macroscopic properties [109,110].

Over the past few decades, a number of models have been proposed for predicting the durability of viscoelastic materials. These models include empirical, statistical, and theoretical approaches. Methods in which polymer degradation is measured under conditions that accelerate its progress, and the obtained kinetic parameters are then extrapolated to predict actual service life, are of great commercial importance. They can be used to develop specifications for quality assurance and control tests, as well as for designing materials with improved properties and performance characteristics.

The use of thermal analysis methods to predict the long-term properties of materials is particularly attractive, as these techniques are fast and the required instruments are widely available. However, applying overly stringent degradation criteria or using inappropriate evaluation criteria can lead to erroneous ageing predictions.

In a typical durability prediction procedure, the activation energy is calculated based on degradation kinetics using the Arrhenius equation. A plot of the logarithm of the time to failure (for example, a 5% mass loss in a thermogravimetric experiment) versus the reciprocal of temperature can be used to predict the time to failure at operating temperatures beyond the experimental range.

Unfortunately, predictions based on such artificial ageing experiments often turn out to be inaccurate, as they inherently depend on the reaction mechanism within the extrapolation range. One possible way to improve the reliability of such analyses is to study materials that have been in service for many years to assess their remaining useful life [132,133,134].

Polymer/POSS composites are characterized by high resistance to ageing processes due to nanoparticles’ unique cage structure, which acts as a protective barrier for the polymer matrix. POSS nanocages can slow down radical-driven oxidation mainly by forming a diffusion barrier (reduced O_2_ penetration and migration of low-molecular degradation products) and, in selected systems, by limiting radical initiation (e.g., through UV absorption). The magnitude of this effect depends on POSS structure, including functionalization, and the kind of polymer matrix. The presence of POSS in composites significantly extends the material’s operational durability. A properly selected nanofiller at a certain concentration can increase resistance to heat, γ radiation, and oxidative agents; however, excessive POSS content can lead to particle agglomeration and loss of the stabilizing effect. The thermal and radiation studies described earlier confirm that POSS nanocomposites exhibit a slower ageing rate and a longer oxidation induction time compared to pure polymers. From a life-cycle analysis (LCA) perspective, the key role of POSS is to extend the composite’s useful life, which translates into lower raw material consumption and reduced waste. However, the durability of these materials depends on the type of POSS functional groups, the degree of filling, and environmental conditions. Although long-term degradation of the silsesquioxane structure itself is possible, the protective effects of POSS significantly reduce the ageing rate and make these composites promising materials for applications requiring high thermal, mechanical, and oxidative stability [104]. For this reason, polymer/POSS composites have become increasingly important in the context of the circular economy and the extended end-of-life (EoL) concept. A longer composite lifespan and improved ageing resistance can help reduce waste generation and improve the utilization of available raw materials. In the case of polymeric materials, it is also crucial to develop strategies for separating and reprocessing composite components, taking into account the impact of nanofillers on mechanical and chemical recycling processes [135]. High thermal and oxidative stability, although desirable during the usage phase, can hinder the biodegradation or depolymerization of the material, requiring the development of innovative methods for recovery or reuse within a closed material cycle. Integrating circular economy principles into composite design allows for the creation of materials with optimized durability that not only meet stringent operational requirements but also align with sustainable development strategies and minimize environmental impact [122].

## 4. Conclusions and Future Outlook

When hybrid and polymer composite structures are exposed to conditions under which thermal degradation and ageing processes may occur, their properties, including mechanical properties, deteriorate and may no longer satisfying to fulfil the initial requirements. Studies of thermal degradation and ageing in complex materials, such as polymer hybrids and nanocomposites, require deep knowledge of the decomposition mechanisms of the polymer matrix, and the effect of the inert or reactive additive [136].

The postulated mechanism and rates of heterogeneous reactions depend on physical factors, which are limited by the system’s viscosity and porosity. Barrier effects influence on the diffusion phenomena and thus contribute to the overall thermal decomposition mechanism. Importantly, the knowledge of the degradation factors and degradation mechanisms is required in order to develop appropriate procedures that permit an evaluation of the properties of the polymer nanohybrids and composites over their lifetime. Such extrapolated lifetime predictions’ reliability considerably increases if key decomposition factors are identified and taken into account.

In practical terms, accelerated ageing tests of polymer composites could be performed using similar procedures and techniques to those for pristine polymers. Specimens after ageing should be investigated by various physico-chemical methods to obtain information on structural and morphological changes induced by heat, UV radiation, and moisture. Among the methods that are applied for studying aged polymeric hybrids and nanocomposites, one can name, again, spectroscopic methods, GPC, XRD, and SEM. Hyphenated techniques, e.g., TG-IR and TG-MS, may add useful information on the mass change and the composition of low-molecular-weight volatiles emitted during the thermal decomposition. Future research efforts will be directed toward the development of heat-resistant and mechanically stable polymeric materials for special applications, e.g., in the aviation industry, and toward intelligent but easy disposable materials with, e.g., temperature sensors as packaging in the food industry. It is also expected that new modelling tools based on AI will be developed; however, it is still of primary importance that reliable structural and morphological data are used in these simulations. POSS additives may significantly improve polymer resistance to thermal, oxidative, and photochemical ageing processes. They create an inorganic barrier that limits access to oxygen and UV radiation and captures free radicals, slowing the degradation of polymer chains. POSS fillers usually increase the degradation temperature, and they can improve the mechanical properties and color stability of the material. A properly selected type and amount of POSS (usually 2–6%) extends the life of polymers, but excess amounts can cause particle agglomeration and property deterioration, leading to performance failure. Properly designed—taking into account circular end-of-life planning—and fabricated polymer/POSS composites can retain their functional properties for a longer period. 

## Figures and Tables

**Table 1:** Autoxidation scheme based on free radical chain reaction theory.

Initiation			
	Polymer	→	$P^{●}+{\;P}^{●}$
Propagation			
	$P^{●}+O_{2}$	→	${POO}^{●}$
	${POO}^{●}+PH$	→	$POOH\;{+P}^{●}$
Chain branching			
	POOH	→	PO●+OH●
	POOH+POOH	→	${PO}^{●}+{POO}^{●}+H_{2}O$
	PH+OH●	→	$P^{●}+H_{2}O$
	$PH\;{+PO}^{●}$	→	$P^{●}+POH$
Termination			
	$P^{●}+{\;P}^{●}$	→	Non-radical product
	$P^{●}+{\;POO}^{●}$	→	Non-radical product
	${POO}^{●}+{\;POO}^{●}$	→	Non-radical product + O_2_

**Table 2:** The aging measurement conditions summary with standards.

References	System/Material	Type of Simulated (Accelerated) Ageing	Ageing Conditions Stated in the Text
[96]	PS + 5% of various POSS	Photo-ageing (UV)	UVB lamps, 55 °C, up to 240 h
[97]	PP/POSS (various organosilicon additives; incl. POSS8, POSS18)	Accelerated laboratory ageing/weathering	Atlas Weather-Ometer Ci4000, according to ISO 4892-2 A1
[98]	Epoxy resins + various POSS	Simulated space environment ageing	Outgassing tests and exposure to atomic oxygen (AO) as the main ageing factor in LEO
[99]	PBO (poly(p-phenylene benzobisoxazole)) + trisilanolisobutyl-POSS	UV ageing (impact on mechanical properties)	UV irradiation under a 400 W lamp, 300–500 nm, exposure times 8 h and 16 h
[100,101]	Ph-POSS@ZIF-8 and Ph-POSS@ZIF-8@PDA@Sponge	“Harsh environmental conditions”—chemical/exposure tests	Tests in acidic, alkaline, and saline solutions; long-term performance: 25 separation cycles
[93]	PU/POSS (moisture-curable PU + epoxy-functional silsesquioxanes)	Thermo-oxidative ageing (yellowing)	The effect is stated: delay of ageing onset from 8.32 h to 20.78 h

## Data Availability

No new data were created or analyzed in this study. Data sharing is not applicable to this article.

## Glossary

POSS	Polyhedral Oligomeric Silsesquioxane
TG-FTIR	Thermogravimetry–Fourier Transform Infrared Spectroscopy
TG-MS	Thermogravimetry–Mass Spectrometry
TGA	Thermogravimetric Analysis
FT-IR	Fourier Transform Infrared Spectroscopy
UV–Vis	Ultraviolet–Visible Spectroscopy
SEM	Scanning Electron Microscopy
EDX (or EDS)	Energy-Dispersive X-ray Spectroscopy
DSC	Differential Scanning Calorimetry
TVA	Thermal Volatilization Analysis
GPC	Gel Permeation Chromatography
XRD	X-ray Diffraction
DMA	Dynamic Mechanical Analysis
Tg	Glass transition temperature
PS	Polystyrene
PP	Polypropylene
PU	polyurethane
ABS	Acrylonitrile–Butadiene–Styrene copolymer.
PBO	poly(p-phenylene benzobisoxazole)
AO	Atomic oxygen
SiO_2_	Silicon dioxide
ZIF-8	Zeolitic Imidazolate Framework-8
PDA	Polydopamine
AAS	Atomic Absorption Spectroscopy
PGMA	Poly(glycidyl methacrylate)
LEO	Low Earth Orbit
LCA	Life Cycle Analysis
EoL	End-of-life
AI	Artificial Intelligence
